# Islet Function in the Pathogenesis of Cystic Fibrosis-Related Diabetes Mellitus

**DOI:** 10.1177/11795514211031204

**Published:** 2021-07-13

**Authors:** Efraim Westholm, Anna Wendt, Lena Eliasson

**Affiliations:** Department of Clinical Sciences in Malmö, Islet Cell Exocytosis, Lund University Diabetes Centre, Lund University, Malmö, Sweden

**Keywords:** CFRD, diabetes, pancreas, islets, β-cells, α-cells, insulin, glucagon, pancreatic insufficiency, crosstalk

## Abstract

Cystic fibrosis-related diabetes mellitus (CFRD) is the most common non-pulmonary co-morbidity in cystic fibrosis (CF). CF is caused by mutations in the cystic fibrosis transmembrane conductance regulator gene (*CFTR*), which leads to aberrant luminal fluid secretions in organs such as the lungs and pancreas. How dysfunctional CFTR leads to CFRD is still under debate. Both intrinsic effects of dysfunctional CFTR in hormone secreting cells of the islets and effects of exocrine damage have been proposed. In the current review, we discuss these non-mutually exclusive hypotheses with a special focus on how dysfunctional CFTR in endocrine cells may contribute to an altered glucose homeostasis. We outline the proposed role of CFTR in the molecular pathways of β-cell insulin secretion and α-cell glucagon secretion, and touch upon the importance of the exocrine pancreas and intra-pancreatic crosstalk for proper islet function.

## Introduction

Cystic fibrosis (CF) is one of the most common autosomal recessive diseases in the world; its global prevalence is estimated to 70 000 to 100 000 affected individuals.^1^ CF is caused by loss of function mutations in the cystic fibrosis transmembrane conductance regulator gene (*CFTR*, also known as the *ABCC7* gene). The CFTR protein is a cAMP regulated plasma membrane anion channel and a member of the ATP-binding cassette (ABC) superfamily of transmembrane transporters.^2^ With its ability to act as a chloride channel as well as to regulate other membrane proteins, CFTR exerts control over the composition of luminal secretory fluids in the lungs, intestines, and the pancreas.^2,3^ Disruptions in this gene lead to disturbed trans-epithelial fluid transport in organ systems such as the bronchial tree, intestines, kidney, male reproductive tract, and pancreatic duct. The most common non-pulmonary co-morbidity of individuals with CF is cystic fibrosis-related diabetes mellitus (CFRD), which usually develops in late adolescence or early adulthood. CFRD is considered a form of diabetes mellitus distinct from type 1 diabetes (T1D) and type 2 diabetes (T2D),^4^ and with a prevalence among adolescent and adult patients of ~20% and ~50%, respectively.^5^

Inadequate insulin secretion dominates CFRD pathophysiology although insulin resistance can occur.^6^ The reason for deficient insulin secretion is complex and both intrinsic and extrinsic reasons have been put forward. It has been suggested that the reduced insulin secretion is a spill-over effect from the damaged exocrine pancreas seen in many CF patients to the pancreatic islets.^7^ However, this view has been challenged as it was shown that exocrine insufficiency and pancreatic damage due to progressive CF did not necessarily correlate with CFRD diagnosis.^8,9^ To add to the complexity, there are data showing that destruction of β-cells is important in CFRD pathogenesis^10^ and other showing no association between CFRD and loss of β-cell mass.^4^ Hence, both an extrinsic mechanism focusing on β-cell destruction due to pancreatic damage and an intrinsic defect in the pancreatic β-cell, attributable to loss of function in CFTR, has been suggested.^11–14^ In favor of an intrinsic pathway behind CFRD is the altered insulin secretion pattern displayed by CFRD patients. In CFRD, as in T2D, the first phase insulin secretion after a meal is absent.^4,15^ However, this cannot discharge that loss of beta-cell mass may also be involved. The etiology of CFRD could thus both be due to damages on the exocrine pancreas with an indirect effect on pancreatic islets; and/or by an intrinsic islet defect through the presence of CFTR in the islet cells.^16^ In this review, we will seek to assess these 2 hypotheses from a cell biological and molecular perspective and give special consideration to how intrinsic effects of CFTR mutations could lead to the altered hormone secretion by β- and α-cells seen in CFRD patients.

## Biochemical and Histopathological Features of CFRD

A typical feature of CFRD is elevated postprandial plasma glucose levels and intolerance to a glucose challenge, rather than elevated fasting glucose levels. Interestingly, abnormal glucose tolerance (AGT), that is inability to lower plasma glucose levels appropriately after a carbohydrate rich meal, is often seen in CF patients regardless of CFRD status.^8,17,18^ Insulin levels are lower in the first phase during an oral glucose tolerance test (OGTT) in CF and CFRD patients.^18–21^ Also, there is an elevated secretion of proinsulin in both CFRD patients and CF patients with impaired glucose tolerance (IGT).^22,23^ Failure to supress initial glucagon secretion following an OGTT correlates with decreased glucose tolerance among CF patients. This suggests that the early elevation of plasma glucose can in part be due to the hyperglycemic actions of glucagon.^24,25^ Even though there is no clear data showing aberrantly high fasting plasma glucagon in CF patients, there are studies in CF patients indicating enhanced hepatic gluconeogenesis, a liver process stimulated by glucagon^26^ and inhibited by insulin.^27^ In T2D, impaired and elevated postprandial plasma glucagon levels exacerbate hyperglycaemia.^28^ At the same time, glucagon release in response to low glucose is impaired in T1D and long lasting T2D increasing the risk of hypoglycemic episodes.^29^ Along the same lines, Kilberg et al^21^ showed that reduced glucagon secretion hours after a meal can lead to hypoglycemic events in CF patients diagnosed with pancreatic insufficiency. The dysregulation of glucagon secretion, with impaired high secretion in the early post-prandial phase^20,24^ followed by a failed counter-regulatory response in the later phase^20,30^ is not well understood.

**Figure 1.**
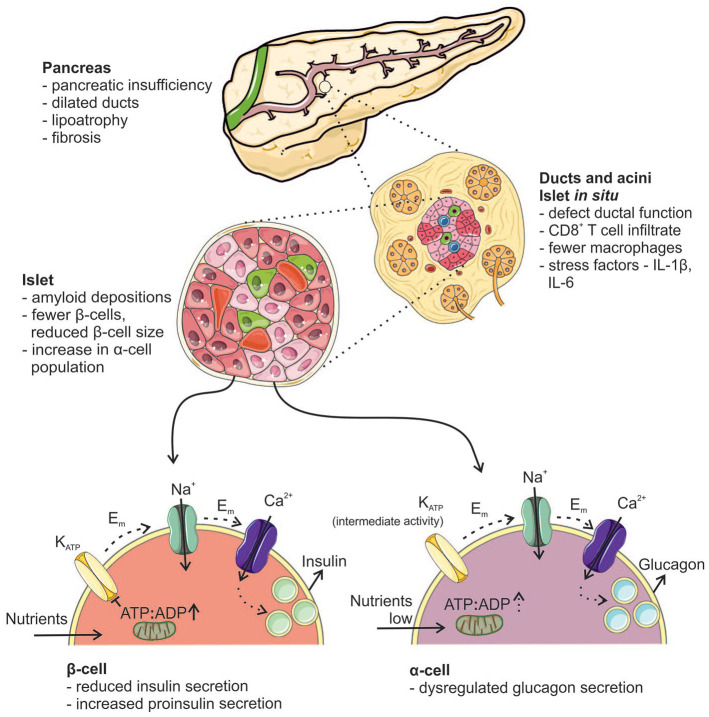
Summary of pathological changes seen in the CFRD pancreas: The gross histopathological changes are listed. Alterations of the ducts and pancreatic islets are outlined together with the changes within the islets themselves. Schematic illustration of the stimulus secretion coupling in the β-cell (lower left) and the α-cell (lower right). Depolarization (*E*_m_) of the plasma membrane opens voltage gated Na^+^ and Ca^2+^ channels, resulting in Ca^2+^ influx and insulin and glucagon release respectively. CD8^+^ T cell, cluster of differentiation 8 expressing T lymphocyte; IL-1β, interleukin-1β; IL-6, interleukin-6; K_ATP_, K_ATP_ channel. The figure was created using Servier Medical templates, which are licenced under a Creative Commons Attribution 3.0 Unported Licence; https://smart.servier.com.

In terms of exocrine pancreatic function, CF patients with pancreatic insufficiency need pancreatic enzyme replacement therapy to digest ingested meals properly. The degree of pancreatic insufficiency correlates with risk of developing CFRD.^31–33^ However, patients without CFRD may also show exocrine damage.^9,34^ An autopsy study of pancreata from young CF patients showed that even before ultrastructural changes of the exocrine pancreas could be detected, there was evidence of altered islet architecture with a reduced number of insulin positive cells and increased number of glucagon positive cells.^35^ Moreover, adolescent CF patients with pancreatic insufficiency display a dysfunctional enteroinsular axis. Secretion of incretin hormones such as gastrointestinal peptide (GIP) and glucagon-like peptide 1 (GLP-1) are lower in CF patients. This is important with regard to CFRD, since incretin hormones modulate both insulin and glucagon secretion. Thus, the disturbed meal uptake, due to pancreatic insufficiency, will likely result in reduced production of incretin hormones affecting the islet cells and their hormone release after a meal.^17,36^ Figure 1 outlines the gross pathological changes to the pancreas seen in CF and CFRD patients alike.

## Evidence for Endocrine Pancreatic Dysfunction Leading to CFRD

### Pancreatic islet histology in CFRD

Islets from CFRD patients have decreased insulin staining compared to age matched controls without CFRD, with a concomitant increase in glucagon staining (Figure 1).^9^ However, there is variability in β-cell mass in post-mortem studies on CF patients.^4^ Prolonged hyperglycemia has been attributed as the cause of reduced β-cell mass as changes in β-cell mass are reversible when blood glucose levels are normalized.^37^ As mentioned, CF patients in general have an abnormal glucose metabolism.^19^

There are conflicting studies on whether CFTR is expressed in islet cells and if it has a role in regulating β-cell physiology. Expression of CFTR in human and rodent β-cells has been investigated by several groups using fluorescence in situ hybridization, immunohistochemistry, and western blot.^10–13,38-41^ In some of the studies CFTR expression was minimal or not at all detected,^10,39,41^ in others a larger proportion of the cells showed CFTR expression.^11–13,38,40^ It is not an easy task to detect CFTR in β-cells. The different results can be due to the use of different antibodies and/or the type of tissue (single β-cells, whole islets, or pancreas tissue) used. Another factor that might play a role is if the tissue material is fresh or has been stored embedded in paraffin for a long time. In one of the latter studies,^38^ Di Fulvio and colleagues made a large effort testing several different antibodies and they came to the conclusion that some but not all CFTR antibodies detect CFTR in pancreatic islets. Their conclusion, which can be agreed on, was that CFTR is expressed in a subpopulation of β-cells. Moreover, we and others have reported the presence of CFTR currents in primary human β-cells by patch clamp.^11,12,38^ In recent years, much attention has been given to islet cell heterogeneity, and that subtypes of β-cells and α-cells have different gene expression and physiological roles.^42,43^ Expression studies are snapshots, and it has not yet been described if a cell stays within a certain expression pattern forever. A recent study describes the likelihood of dynamic changes in expression over time and how β-cells take turns to have different set-ups, just as migrating birds with a leader that changes.^44^ With this in mind, CFTR is most likely expressed in a subpopulation of β-cells that dynamically changes.

### CFTR and β-cell physiology

The stimulus secretion coupling of β-cells is closely linked to regulation of the plasma membrane potential.^45^ The current consensus pathway in brief (Figure 1): metabolism of glucose or other nutrients elevates the cytosolic ATP:ADP ratio, resulting in closure of K_ATP_ channels and a slight depolarization. The depolarization is then augmented by opening of T-type Ca^2+^ channels which leads to sequential opening of voltage gated Na^+^ channels, L and P/Q-type Ca^2+^ channels, furthering the membrane depolarization, and Ca^2+^ influx. The rise of intracellular Ca^2+^ enables insulin granule exocytosis.^46^ In addition, Cl^−^ currents have been recorded in insulin secreting cell lines and have been proposed to contribute to β-cell membrane depolarization.^47,48^ It is notable that although Cl^−^ currents often are hyperpolarizing in neurons^49^ this is not the case in β-cells where the intracellular Cl^−^ concentration has been suggested to be kept high by the presence of Cl^−^ transporters.^48^ This will lead to efflux of Cl^−^ though Cl^−^ channels at negative membrane potentials. Therefore, activation of a Cl^−^ channel would depolarize the β-cell and increase the probability of insulin release. Indeed, application of GABA depolarized human β-cells, through binding to GABA_A_-receptors, and increased electrical activity.^50^ Other Cl^−^ channels suggested to be present and contribute to insulin secretion are VRAC (Volume-regulated anion conductance) and ANO1 (Anoctamin 1).^47,48,51^

Given its known Cl^−^ channel activity, several studies have been conducted with the explicit aim to elucidate whether CFTR is involved in regulating the plasma membrane potential in β-cells and thereby influence insulin secretion.^11,12,14^ Guo et al^12^ recorded cAMP-dependent Cl^−^ currents in the plasma membrane of primary β-cells from WT mice but not in a mouse model harboring the ∆F508 mutation in *CFTR*. By administering Lumacaftor, a corrector for the ∆F508 mutation, CFTR channel activity, and insulin secretion was restored in islets from ∆F508 mice.^12^ In a study by Di Fulvio and co-workers performed in rat β-cells a CFTR Cl^−^ current was measured in 33% of the cells.^38^ Finally, we have measured the presence of a CFTR Cl-current in primary human β-cells.^11^ Thus, it is likely that CFTR has a role in the depolarization of β-cells. Measurements of action potential firing in presence of a CFTR inhibitor would definitely prove if this is the case.

Apart from being an ion channel CFTR has been suggested to be a regulator of other proteins,^52^ including other ion channels such as ANO1.^53^ We have investigated this possibility in human and mouse β-cells using insulin secretion measurements, the patch-clamp technique, and capacitance measurements to measure exocytosis.^11^ In a series of experiments performed in human islets cAMP-amplified glucose-stimulated insulin secretion was attenuated with the simultaneous addition of the CFTR channel blocker GlyH-101. Moreover, a blocker of ANO1, AO1, reduced glucose- and cAMP-stimulated insulin secretion to the same extent, and addition of GlyH-101 in the simultaneous presence of ANO1 did not cause an additive decrease. From these observations we have suggested that an additional role for CFTR is to effect insulin secretion through downstream regulation of ANO1.

**Figure 2.**
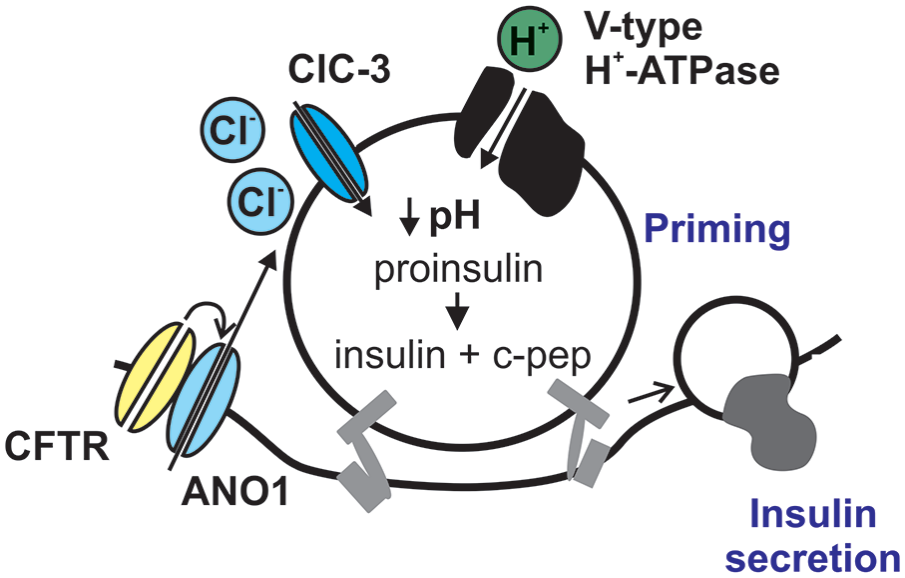
The role of CFTR in insulin granule priming: CFTR regulates ANO1, resulting in an influx of Cl^−^ near the insulin granule. This provides ClC-3 with Cl^−^ ions to pump in to the granule. Cl^−^ is a necessary counter ion for H^+^, which is pumped in by V-type H^+^-ATPase. The granule pH is lowered, permitting cleavage of proinsulin to insulin and c-peptide. Insulin secretion occurs via granule fusion to the plasma membrane. Abbreviations: CFTR, cystic fibrosis transmembrane conductance regulator; ANO1, Anoctamin 1; ClC-3, chloride channel-3.

In the same study,^11^ we measured reduced increase in membrane capacitance in the presence of GlyH-101. Analyses of the exocytotic response suggested reduced priming of insulin granules. Priming is a process by which the insulin granules become release ready. Lowering of granular pH though acidification is part of the priming process.^54^ One of the functions that require a low pH is the cleavage of proinsulin into insulin and c-peptide by the prohormone convertases PC1/3 and PC2.^55^ The acidification of granules requires the simultaneous pumping in of protons (by V-type H^+^-ATPase) and influx of Cl^−^ as counter ions (suggested to enter via chloride channel-3, ClC-3, a Cl^−^/H^+^ antiport).^54,56,57^ We propose that CFTR, through its activation of ANO1, supplies ClC-3 with the necessary Cl^−^ ions for this acidification (Figure 2). Indeed, in the ∆F508 mouse β-cell pH is elevated, priming is disturbed, and there is an increased secretion of proinsulin and a decreased secretion of c-peptide.^58^

The data showing impaired insulin secretion in both primary human β-cells, and animal models with the ∆F508 mutation seems to reflect the observed disturbed first-phase insulin release in CF and CFRD patients alike.^8,17^ Abnormal and impaired glucose tolerance is seen in CF patients as well.^18,19^ Additionally, patients with CF have a skewed secretory ratio of proinsulin:insulin,^22,23^ which could be attributed to the proposed role for CFTR in insulin granule priming.

In all, currently there exist data pointing toward a role of CFTR in membrane depolarization as well as in insulin granular priming and exocytosis. Thus, it is most likely that a CFTR dysfunction in β-cells contributes to the altered glucose homeostasis in CFRD.

### CFTR and α-cell physiology

Glucagon, secreted by the α-cells in the pancreatic islets, is the main hormone for elevating plasma glucose and act in a counter-regulatory fashion to the hypoglycaemic effects of insulin. The stimulus secretion coupling in α-cells is not well understood. It is suggested that intermediate activity of the K_ATP_ channel at low blood glucose levels causes enough depolarization for voltage-gated Na^+^ and P/Q-type Ca^2+^ channels to open, providing Ca^2+^ ions necessary for glucagon granule fusion (Figure 1).^59^ The resting conductance is lower in both rodent and human α-cells and is attributed to the intermediate activity of the K_ATP_ channel.^60,61^ This results in a different response to glucose in comparison to β-cells.

CFTR has been detected by immunohistochemistry in mouse, rat, and human α-cells.^38,40,62^ Like in β-cells there are also some studies against the presence of CFTR in α-cells.^10,39^ In our hands, inhibition of CFTR increased cAMP potentiated glucagon secretion in human islets.^62^ Elevated level of plasma glucagon was measured after an intra-peritoneal glucose tolerance test in the ∆F508 mouse model, supporting that functional CFTR negatively regulates glucagon secretion.^63^ Current available data suggests that CFTR hyperpolarizes the α-cell membrane potential.^62,63^ Similar hyperpolarizing effects has been shown after stimulation of GABA_A_ Cl^−^ channels in α-cells.^64^

There is data from rodent models supporting that CFTR may be more expressed in α-cells compared to β-cells^40^ and that functional impact of dysfunctional CFTR may therefore be more important in this cell type.^62,63^ However, the current lack of understanding of α-cell secretory and membrane physiology in general makes the translation of this effect in CF and CFRD patients more difficult. There is only modest evidence for increased glucagon secretion in CF and CFRD patients.^26^ Rather, the only established secretion abnormality is failed counter-regulatory secretion of glucagon in late phases of OGTT trials, even though there may be some aberrant increased secretion in the early phase.^20,21,30^

In this context it is interesting to note that glucagon modulates insulin secretion through paracrine interactions within the islet. This is important to keep the correct glucose set point. Indeed, it has been suggested that low levels of glucagon release (which might be difficult to measure) has mainly paracrine effects while higher levels are necessary for systemic effects.^65^ Nevertheless, further studies are needed to elucidate α-cell function in CFRD patients, both in fasting and during OGTT.

## Evidence for Exocrine Pancreatic Disease Leading to CFRD

### CFTR in ductal cell physiology

The central concept explaining how defective CFTR in the exocrine parts of the pancreas may lead to CFRD, focuses on how the mutation of the protein leads to dysfunctional enzyme secretion, local inflammation, and effects thereof on the islets. It also highlights how ductal obstruction can lead to further inflammation and auto digestion.^16^ The cell type of the pancreas that has the highest expression of CFTR are the ductal cells.^42^ It is established that dysfunctional CFTR in ductal cells leads to lower luminal pH and disrupts the function of the secreted digestive enzymes, leading to pancreatic insufficiency.^33,66,67^

Paracrine signals from ductal epithelium have been proposed to play a role in islet physiology.^68^ Ductal cells produce TGF-α and regulate islet differentiation.^69^ TGF-α is overtly upregulated in ductal cells in the context of chronic pancreatitis,^70^ showing that intra-pancreatic crosstalk between exocrine and endocrine tissue could be important in the development of CFRD.

In one study, systemic administration of CFTR inhibitor 172 (CFTRinh172) in healthy mice reduced β-cell size and thereby islet area. These islets also displayed a reduction in insulin content.^71^ Though, if this effect of CFTRinh172 was due to effects on islet cells or ductal cells is difficult to ascertain. In general, there are very few studies^39,68^ directly focused on investigating paracrine interactions between exocrine and endocrine tissue in the pancreas of CF and CFRD patients, and we concur that more research is needed in this area.

### Inflammation and Immune Cell Infiltrate in CF Islets

Primary human ductal cells produce and release TNF-α upon exposure to cytokine IL-1β, which negatively affects β-cell survival.^72^ In islets from patients with both CF and CFRD, there is an elevated IL-1β immunoreactivity and an increased T cell presence, including CD8^+^ T cells.^35,73,74^ Macrophages, specifically anti-inflammatory M2 type, are important in β-cell regeneration by inducing SMAD7 in β-cells. This induction inhibits actions of pro-inflammatory cytokines of the TGFβ superfamily on the β-cells.^75^ Macrophages are also important regulators of angiogenesis in the islets and protect from islet cell loss in mice.^76^ The importance of macrophage and β-cell crosstalk in β-cell regeneration in both T1D and T2D is well researched.^77^ Macrophages are greatly decreased in pancreatic islets from adult patients with CF.^74^ However, the role of macrophages in CF islet pathology and inflammation is not established. In general, the immune cell infiltrate in CF islets is different than that of T1D islets.^33,35^ CF islets do not display the autoimmune destruction seen in T1D islets. Intriguingly though, CF islets do show amyloid depositions similar to those seen in islets from T2D patients.^4^ What is clear is that local inflammation can negatively affect β-cell function in the CF pancreas.^78^

Regarding inflammatory cytokines in CF animal models, Sun et al^39^ demonstrated that islets from neonatal CF animals had lower glucose stimulated insulin secretion and a lower insulin content compared to WT controls. The researchers measured differential secretion of interleukins, and from CF islets IL-6 secretion was elevated. Treatment of WT islets with IL-6 recapitulated a similar phenotype of impaired insulin secretion as in the CF islets.^39^ This finding, together with the altered immune cell infiltrate, supports the role of inflammation and production of intra-islet cytokines that affect β-cell function in CFRD (Figure 1).

## Conclusions and Future Perspectives

CFRD develops in patients already afflicted by CF. These patients have often displayed an abnormal glucose tolerance already at diagnosis.^6^ What is striking is how the prevalence increases with age, suggesting that the CFTR mutations over time may lead to CFRD.

The altered islet hormone secretion in CFRD patients, in particular after meal intake,^17^ shows that normal islet cell physiology is disrupted. A subpopulation of β-cells express CFTR,^38^ and several in vivo and in vitro studies have shown that pharmacological manipulation of CFTR in both β-cell and α-cells disrupt hormone secretion.^11,12,62,63^ Some results available so far suggest effects on α- and β-cell membrane potential, other data like the differential secretion of proinsulin is indicative of that CFTR has a role in insulin granule priming.^22,58^ Taken together, this suggests that dysfunctional CFTR in islet cells alters glucose homeostasis, and that multiple cellular functions can be affected. Yet other data point toward that insulin and glucagon secretion is disturbed in CFRD due to dysfunctional CFTR in the exocrine pancreas^10,39^. One also need to consider that the rodent CF-models do not develop spontaneous hyperglycemia^79^ as do humans. Therefore, caution should be taken when making hypothesis on human CFRD pathophysiology based on these models. This is why the available complementary human islet data^11,12,38^ is extremely valuable.

In conclusion, from what is currently known, a single model cannot explain CFRD. There are data in line with extrinsic deterioration of β-cells, but also data on an intrinsic decline of β-cell function in CFRD. Even though some studies cannot prove the presence of CFTR in the endocrine pancreas, other data, including ample electrophysiological data presented above, show the presence of CFTR in islet cells, and suggest that both β-cell membrane potential and exocytosis is regulated by CFTR. Our proposal is that dysfunctional β-cells, and α-cells, lead to abnormal glucose tolerance which is exacerbated over time by exocrine influence. Disturbed pancreatic crosstalk between exocrine and endocrine compartments likely plays a significant role in β-cell dysfunction. Currently, the least researched facet of CFRD is how paracrine signals from ducts and acini may further impair β-cells, and here more studies are needed. Hopefully, the CFRD incidence can be reduced and lifespan for CF patients can be improved if we manage to halt the deteriorative process occurring in the endocrine and exocrine pancreas of CF patients.
